# Improving maternal and child nutrition services in community based health planning and services zones in the jirapa municipality of northern ghana-challenges and strategies: the perspective of community health officers

**DOI:** 10.1186/s40795-024-00848-8

**Published:** 2024-06-14

**Authors:** Francis Xavier Tengepare, Dennis Chirawurah, Stephen Apanga

**Affiliations:** 1 Catholic Diocesan Health Directorate, Upper West Region, Wa, Ghana; 2 Department of Environmental and Occupational Health, School of Public Health, University for Development Studies, Tamale, Ghana; 3 Department of Community Health and Preventive Medicine, School of Medicine, University for Development Studies, Tamale, Ghana

**Keywords:** Community health officers, Jirapa municipality, Maternal and child nutrition services, Community-based health planning and services

## Abstract

**Background:**

The Community-based Health Planning and Services (CHPS) initiative plays a key role in delivering maternal and child health nutrition services in Ghana. This study explored bottle necks hindering the delivery of maternal and child nutrition services at CHPS zones and searched for strategies to address them from the perspective of community health officers in rural Northern Ghana.

**Methods:**

An exploratory qualitative cross-sectional study design using key informant interviews involving the municipal nutrition officer and Community Health Officers (CHOs) from eleven CHPS zones was carried out in April 2019. Manual data analysis was done using the framework analysis approach in qualitative data analysis for applied policy research.

**Results:**

This study identified challenges of maternal and child nutrition services in the Jirapa municipality to be municipal health directorate and CHPS zone based in nature. Municipal health directorate based challenges were inadequate logistics/medicines; lack of staff training; lack of supervision/monitoring; and inadequate financial support/motivation/incentives. CHPS zone based challenges were lack of planning activities by staff; inadequate home visits; lack of commitment by staff; and lack of community meetings/engagements. Proposed strategies to address municipal health directorate based challenges included adequate provision of logistics/medicines; frequent training of staff in maternal and child nutrition related issues; frequent supervision/monitoring activities from the municipal health directorate; and providing financial support/motivation/incentives at the CHPS zones. Proposed strategies to address CHPS zone based challenges were planning of activities; improved home visits; increased commitment towards delivering maternal and child nutrition services; and frequent community meetings/engagements.

**Conclusion:**

In order to improve maternal and child nutrition services at CHPS zones, there is the need to address certain systemic challenges at both the municipal or district health directorate and CHPS zones levels of the primary health care system. It is recommended that, the Municipal Health Directorate; the Municipal Health Management Team; the Municipal Assembly and all relevant stakeholders involved in improving maternal and child nutrition services at the community level, actively engage CHOs to help address the systemic challenges.

**Supplementary Information:**

The online version contains supplementary material available at 10.1186/s40795-024-00848-8.

## Background

Improving maternal and child nutrition is a major public health challenge which continue to receive much global attention as a developmental goal through the Sustainable Development Goals (SDG) and Universal Health Coverage (UHC) drive [1–4]. Poor maternal nutrition has been found to have lifelong consequences on the health of a mother and her child’s overall growth and development. It is estimated that nearly 3.1 million children die annually in Low and Middle-Income Countries (LMICs) from nutrition-related events such as stunting, small for gestational age and wasting among others [5].

Maternal and child malnutrition still remain high in Ghana with about 20% of children under 5 years suffering from stunted growth [6, 7] and 45% of pregnant women suffering from anemia [7]. Recent data on stunting indicates northern Ghana as the most affected with regional variations of 37.1%, 35.8% and 25.1% for the Northern, Upper East and Upper West regions respectively [3, 8] and thereby requiring the needed attention from all stakeholders.

Although health systems strengthening strategies at all levels of the health care system have been found to be critical towards achieving UHC in most countries, nutrition interventions at the Primary Health Care (PHC) level have been established to play a major role in both disease prevention and health promotion activities resulting in a reduction in the burden of diseases and thus contributing towards the realization of the SDGs [1].

The Community-based Health Planning and Services (CHPS) initiative in Ghana serves as the pillar of its PHC system. The CHPS concept which was initially piloted in the Kassena-Nankana and Nkwanta districts of the Upper East and Volta regions respectively proved that engaging a resident nurse in a community, involving traditional leaders and community members in the provision and management of health care services significantly reduced maternal and child mortalities including increased uptake of family planning services [9]. The CHPS concept which was scaled up as a national program in 2001 was subsequently revised and re-launched in 2016 to address certain policy and systems level challenges related to leadership, technical direction, supervisory support, planning and budgeting. The operational framework of the CHPS initiative was to provide essential PHC services and health education within demarcated geographic areas often referred to as CHPS zones which are staffed by resident Community Health Officers (CHOs) that operate from small service delivery posts known as CHPS compounds. These CHOs are supported by community volunteers made up of both Community Health Volunteers (CHVs) and Community Health Management Committees (CHMCs) who are responsible for mobilizing community resources and related activities [10, 11].

A critical component of the revised CHPS policy was to help ensure the implementation of the National Nutrition Policy (NNP) at the community level. The main objective of the NNP is to educate people about the importance of investing in nutrition, guide the implementation of evidence-based nutrition interventions and promote healthy lifestyles and appropriate dietary habits [3, 12]. However, despite the implementation of this policy in addition to interventions such as the Maternal and Child Health and Nutrition Improvement Project (MCHNP) [4]; Maternal and Child Survival Program Ghana (MCSP) [13]; and adopting healthy maternal and child survival practices in rural Ghana [14] which were all aimed at improving maternal and child nutrition, maternal anemia and stunting are still high in the Upper West Region of Ghana [6]. Jirapa has one of the worse maternal and child nutritional status in the Upper West Region in terms of anemia in pregnancy and stunting according to annual performance review reports from the nutrition unit of the Jirapa Municipal Health Directorate (MHD) [15]. The persistent state of poor maternal and child nutrition in the municipality notwithstanding the numerous interventions through the CHPS zones, might point to some inherent systemic challenges which need to be investigated. This study therefore explored challenges of maternal and child nutrition service delivery and strategies to address them at the CHPS zones from the perspective of community health workers in the Jirapa municipality of rural northern Ghana.

## Materials and methods

### Study settings

The Jirapa municipality is located in the north western part of the Upper West Region of Ghana with its capital being Jirapa which is peri-urban and the rest being mainly rural. The municipality has 137 communities with 2 of them sharing borders with Burkina Faso and 15 of them hard to reach during the rainy season. The municipality is located within the Guinea Savanna climatic Zone and experiences one season of rainfall and a long dry spell. The main economic activity of the people is agriculture with close to 90% of the people engaged in crops and animal farming. The projected population of the district for 2019 by the MHD based on the 2010 population and housing census was estimated to be 104,720 people at a growth rate of 1.9 [15].

Records on nutrition indicators from the nutrition unit of Jirapa MHD indicates that the municipality has one of the worse maternal and child nutritional status in the region. The health infrastructure is made up of 45 health facilities consisting of one hospital which serves as the main referral point; one polyclinic; seven health centres; and thirty six CHPS zones [15].

### Study design

This was an exploratory cross-sectional qualitative study carried out from 5th to 30th April, 2019 using Key Informant Interviews (KIIs). Key Informant Interviews (KII) are in-depth interviews that collect data from individual experts requiring careful selection of subjects to gather input from individuals considered to be the most knowledgeable people on the subject [16]. We used KIIs because the participants of this study had received specific training on maternal and child nutrition service delivery at the CHPS zone level.

### Selection of participants

The heads of CHPS zones (15) and the municipal nutrition officer (1) were selected as key informants for the in-depth interviews. These participants were purposively selected because they were deemed to be the key stakeholders in terms of managing maternal and child nutrition services in the health care system at this level of health care.

### Data collection

A graduate level research assistant assisted in the data collection after receiving one week of training on the study protocol and qualitative data collection with emphasis on conducting KIIs and qualitative data analysis. An in-depth interview guide (see supplementary file) that contained questions related to challenges and proposed strategies to improve maternal and child nutrition services was used to conduct the interviews. The questions of the in-depth interview guide were based on two main thematic areas: MHD related and CHPS zone related. All interviews were conducted in English language and audio-recorded for later transcription after pre-testing the tool in one CHPS zone which was not part of the final study. At pre-testing, the in-depth interview guide was used to conduct a 45 min interview of the in-charge of a CHPS zone which was not included in subsequent data collection. This helped the researchers to fine tune the in-depth interview guide before final deployment for data collection. Using the data saturation approach [17, 18], saturation was achieved after 12 in-depth interviews due to informational redundancy-as subsequent participants were repeating the information previous participants had already provided. Each interview lasted between 30 and 50 min.

### Data analysis

The audio-recordings were transcribed verbatim into texts for analysis. Manual data analysis was done using the framework analysis approach in qualitative data analysis for applied policy research as outlined by Jane Ritchie and Liz Spencer [16, 19]. Framework analysis is a qualitative method that is best suited for applied policy research and it is better adapted to research that has specific questions, a limited time frame, a pre-designed sample (e.g. professional participants) and a priori issues (e.g. organizational issues) that need to be dealt with [16] hence making it the most appropriate for this study. A code book was generated a priori with predetermined themes as logistics/medicines; training; supervision/monitoring; financial support/motivation/incentives and community meetings/engagement. After thoroughly reading through the transcribed transcripts by all three researchers, new emerging themes were identified and the code book updated. Two (Dennis Chirawura and Francis Xavier Tengepare) researchers then independently coded the transcripts according to the thematic areas and extracted quotes. All three researchers then met together to review the coding and analyzed the data using both deductive and inductive data analysis. The deductive analysis allowed for data organization into a priori themes (issues) whist the inductive analysis allowed for new emerging themes within the data. Where there was a disagreement between the first two coders, all three researchers collectively agreed and settled one code.

## Results

A total of twelve key informants were interviewed in this study comprising the municipal nutrition officer and eleven CHOs (heads or in-charges) from various CHPS zones. The average age of the participants was 32 years with other characteristics shown in Table 1 below.

**Table 1:** Characteristics of participants

Characteristics	Gender	
	Male	Female
Number (%)	7 (58)	5 (42)
Average age (years)	32	33
Average duration of stay in CHPS zone (years)	5	4

Source: Field survey 2019

### Thematic areas

The main thematic areas identified are presented in two sections comprising challenges and strategies pertaining to both the MHD and CHPS zones and outlined in Table 2.

**Table 2:** Emerging main themes and sub-themes

Main themes	Sub-themes	
	Challenges	Strategies
Municipal Health Directorate related challenges and strategies	1. Inadequate logistics/medicines 2. Lack of staff training 3. Lack of supervision/monitoring 4. Inadequate financial Support/motivation/incentives	1. Provision of adequate logistics/medicines 2. Frequent training of staff 3. Frequent supervision/monitoring 4. Provision of financial support/motivation/incentives
CHPS zone related challenges and strategies	1. Lack of planning by staff 2. Inadequate home visits 3. Lack of commitment by staff 4. Lack of community meetings/engagements	1. Good planning of activities 2. Improved home visits 3. Increased commitment by staff 4. Frequent community meetings/engagements

Source: Field survey 2019

### Challenges and strategies relating to the municipal health directorate

#### Logistics and Medicines

Inadequate logistics was identified as one of the major challenges to the delivery of nutrition services. Key amongst this challenge were lack of transport in terms of motor bikes; essential medicines such as zinc tablets, oral rehydration salts (ORS) and vitamin A; and maternal and child health record books for monitoring. The lack of logistics according to some participants was not only at the level of the CHPS zones but also at MHD and sub-district levels.

Some examples of statements from participants concerning logistics include:

The major challenge is the means of transportation. At times you want to go to the community to do some activities but the transportation is always a problem. Sometimes too the fuel they provide is always limited and cannot be used for the whole month if even you are using your own transportation (KII with participant B).

In my CHPS zone, we don’t have maternal and child health record books and Vitamin A. Sometimes we don’t get them and as of now they are not in the system. I mean Vitamin A red and blue (KII with participant B).

All participants were of the opinion that the MHD which is the administrator of health care delivery at the CHPS zone level, do everything possible within its means to provide the necessary logistical support for effective delivery of maternal and child nutrition services. One participant buttressed this point by saying that;

The district [MHD] needs to take up child health promotion week and other programs that support child health and nutrition seriously. They should make sure that the logistics for such exercises are provided for the work to be done. And also make sure that the logistics needed for growth monitoring and other child nutrition programs are always available. As of now we need vitamin A to run the services and yet it is not there. So they should make sure that the logistics are provided and do follow-up and monitor to make sure that they are used effectively (KII with participant K).

Some participants also intimated the need for the MHD and other stakeholders to support the CHVs and CHMCs with logistics such as bicycles, raincoats and microphones amongst others to aid in their functions. One participant had this to say concerning supporting the CHVs.

For me I think megaphones should be provided to the volunteers. Sometimes they are not able to enter every household but with the megaphones they can stand at one place in the community and deliver health messages (KII with participant C).

#### Training

Lack of regular training on nutrition for staff at the CHPS zones was found to be a challenge in delivering maternal and child nutrition services in the municipality.

The need for regular training for both the CHOs and CHVs on maternal and child nutrition related issues was mentioned by 33% (*n* = 4) of the participants as a strategy to address this challenge.

I think the district should organize training especially on maternal and child nutrition because most CHOs are not trained on maternal and child nutrition but if they organize quarterly training for us, I think we can improve on most of the service delivery we are carrying out in the communities. So they should also organize workshops and meetings for us to improve on maternal and child nutrition health. The people are very ignorant on maternal and child nutrition issues. The Nutritionists at the MHD should be involved in all activities related to nutrition because they know more. So they should come to the CHPS zones and conduct nutrition education (KII with participant J).

More staff should be trained on maternal and child nutrition. Most of the staff have only received on the job training but when you train them formally, I thing that one will be a little bit different from on the job training. Constant refresher training should also be given to the CHOs (KII with participant I).

Similarly 33% (*n* = 4) of the participants were of the view that considering the critical role CHMCs play in community actions and activities [11], they needed some training on maternal and child nutrition related issues as well. A participant at one of the CHPS zones had this to say regarding the need to train CHMCs.

We would have been happy if more training could be given to them [CHMCs]. They are there in the community. If more training is given to them they can help in our education to the people. So yes training of the community health committee members because they help the nurses a lot (KII with participant E).

#### Financial support/Motivation/Incentives

Inadequate and sometimes a complete lack of financial support for maternal and child nutrition activities was identified as a major challenge by about 42% (n = 5) of the participants. Whereas some participants indicated that they needed monthly stipends to fuel their motor bikes for home visits, others said they needed funds to repair their broken down motor bikes. Participants were of the opinion that either a monthly or quarterly imprest from the MHD for their activities will help improve the delivery of maternal and child nutrition services. They also indicated that such an imprest will enable them purchase basic stationary items such as pencils and books for their activities.

We used to carry out a whole lot of activities in nutrition but now we can’t do them because there is no money” (KII with participant L).

So monthly stipends like imprest if even it is just GH100 every month, so that you can use it to buy pencils, pens, books and other things to be able to run your activities very fast. You can’t do all these things without thinking that you will not spend anything. At least monthly stipends and imprest from the mother-sub account will do” (KII with participant A).

Some participants were of the view that motivating staff to stay and work at the CHPS zones is not only about financial rewards or incentives but also about the MHD recommending staff for further studies to improve their skills. A participant in one of the CHPS zones made this point clear by saying that;

Those CHOs that are at the interior if even they are not giving anything, at least they should always recommend them to go to school and that will motivate others to also feel that being in the CHPS compound you are not wasting your time there (KII with participant B).

The participants also recognized the fact that they do not work alone at the CHPS zones and therefore any form of motivation should also be extended to the CHVs and CHMCs. Majority (58%) of the participants opined that the CHVs should be motivated by providing them with monthly stipends, gifts and soap amongst others. 17% of the participants believed that motivating CHMCs will go a long way in improving maternal and child nutrition services at the community level.

I know that they are volunteers and they are supposed to do it free of charge but at least annually, they [MHD] can always do something to support them. Even a bar of soap to each volunteer will let them contribute better and they will know that we appreciate their efforts (KII with participant F).

They [CHMCs] are the first people that we meet before we do anything. We discuss with them and tell them what our challenges are and we sit as a house. Looking at their work, it is similar to the volunteers but for them, they also implement health policies in the community and so we can give them support as well. We can also give them some T-shirts just to identify them in the community and they will be happy. They also need motivation (KII with participant D).

#### Supervision/Monitoring

Lack of supportive or supervisory visits and monitoring from the district and sub-district levels was found to be another major challenge towards realizing maternal and child nutrition services at the CHPS zones. Over a third (42%) of the participants identified this as one of the challenges they face at their CHPS zones. Some participants however believed that the lack of supervision and monitoring was as a result of the inadequate funding and logistics for such activities.

The participants therefore reiterated the need for the MHD to do everything possible to embark on regular supportive or supervisory visits to the CHPS zones as this will put them on their toes or spur them up. Participants also suggested that these visits could be planned in such a way that the will coincide with durbar days where nutritionists and other experts could assist the CHOs and CHVs to educate community members on maternal and child nutrition topics.

Some examples of these sentiments are stated below:

I have been saying it over and over that effective supervisory monitoring of staff at the various CHPS zones is so important. This will let you see what they [staff] are doing and then you can also add your voice. But when a nurse is at the community and you don’t visit or do anything, the person too will relax (KII with participant E).

The district should do regular supportive visits to the CHPS zones. If I know that the district is coming to my CHPS compound all the time, I will always make sure that am available and do my work. The district [MHD] should be available to all CHPS compounds and more especially to those they think are not performing (KII with participant F).

I think their [MHD] support visits will help us. So when we organize a durbar and we ask for their support and they show up and participate in it, it will help us. Because sometimes the communities don’t take we alone serious but when they hear that somebody is coming from the office, they are eager to come and hear what the person has for them (KII with participant C).

### Challenges and strategies relating to CHPS zones

#### Home visits

Lack of regular home visits by CHOs and CHVs was reported by 25% of the participants as a challenge to improving the delivery of maternal and child nutrition at the CHPS zones.

Participants stressed the need for CHOs to strengthen their home visits responsibility and take advantage of such visits to educate mothers on maternal and child nutrition related activities. Some participants also said that regular home visits could serve as a good avenue for them to embark on food demonstration activities since some mothers still do not know how to prepare nutritional foods.

The CHOs should do weekly home visits and carry out education concerning nutrition to the mothers at home and do individual counseling for nursing mothers and pregnant women. The CHO can also do food demonstration with the mothers (KII with participant A).

Sometimes we also do food demonstration. Some mothers don’t know how to organize some components of food together so we do organize food demonstrations where mothers learn how to prepare various and different foods for their children (KII with participant D).

#### Community meetings and engagement

One of the challenges identified by the participants was the lack of regular meetings between CHOs and community members. A cross Sect. (25%) of the participants suggested the need for regular meetings especially through community durbars as an effective way of improving maternal and child nutrition services. They were also of the view that such meetings could serve as good platforms for carrying out educational campaigns on maternal and child nutrition related issues at the community level.

For the CHOs, I think we have to hold monthly meetings to educate the community through our durbars, child welfare clinics and home visitation in order to improve and strengthen maternal and child health nutrition and promotion (KII with participant G).

So if you have mother-to-mother support groups, you need to hold meetings with them and to do community nutrition assessment to see whether the community members are really feeding well. You should also conduct durbars and educate them on all these and support pregnant women (KII with participant K).

Some participants also identified inadequate community engagement as a challenge in carrying out their duties and therefore admonished the need for CHOs to actively engage the communities in their lines of duty as this could help them mobilize local resources to assist in their activities. A CHO in one of the CHPS zones said that;

Serious community engagement; If you engage the community on some of these [maternal and child nutrition services] issues, they will help you to at least arrange local resources to be able to run your activities (KII with participant C).

#### Planning

Lack of proper planning by some CHOs as to how to carry out their activities at CHPS zones was mentioned as a challenge by some participants. The participants advocated for the need for CHOs to plan their work schedules in a way that will include improving maternal and child nutrition services in their activities.

You [CHO] need to plan and schedule your work in such a way that nothing is left out when it comes to supporting community nutrition initiatives such as the Infant and Young Child Feeding initiative and the rest (KII with participant K).

#### Commitment

Participants expressed their worry about some CHOs not showing enough commitment when it comes to providing maternal and child nutrition services at the CHPS zones although they attributed this to inadequate motivation from the health authorities. They therefore called for an increased level of commitment from the CHOs towards maternal and child nutrition services and to see this as part of their core duties whether they are motivated or not.

They [CHOs] should just be committed to the job. Once they are committed to the job, they can do better. We shouldn’t wait for somebody to motivate us in cash or in kind before. So generally, I think everything is just about commitment and yes if we all take it up as a serious challenge, we can all make a difference (KII with participant F).

## Discussion

This study explored challenges associated with delivering maternal and child nutrition services at the CHPS zones level of the PHC system and strategies to address them from the perspective of community health care workers who are responsible for delivering such services in the Jirapa municipality of rural northern Ghana.

The participants identified the main challenges to delivering effective maternal and child nutrition services to be attributable to the municipal health directorate and the CHPS zones themselves. Attributing some of the challenges to the municipal health directorate is not surprising because the district or municipal health directorate through the district or municipal director of health services has overall responsibility of managing the CHPS zones. Direct supervision of the CHO is however the responsibility of the officer in charge of a health centre in the sub-district who also reports to the district or municipal director [12]. However, the fact that participant also acknowledged challenges from their own end meant that they would be part of the solution to improving maternal and child nutrition services and not shift all responsibilities to the district or municipal health directorate.

Inadequate logistics in the form of transport and stationary and the lack of nutrition related medicines for delivering maternal and child nutrition services at CHPS zones was identified by participants to be a major challenge in the Jirapa municipality. Other studies have equally reported lack of logistics as a major challenge in the operations of the CHPS initiative in Ghana. Many of such studies have pointed out the lack of essential medicines as major constraints with a few mentioning inadequate transport [20–22]. Since the CHOs do not operate in isolation but work together with the CHVs and CHMCs, participants expressed the need for some logistics to be extended to these volunteers to enable them assist in delivering nutrition services to mothers and children in the communities.

Participants also bemoaned the lack of training for CHOs, CHVs and CHMCs that are specifically targeted at improving maternal and child nutrition at the CHPS zones. As suggested by some participants, in-service training of the CHOs, CHVs and CHMCs by the district or municipal nutritionists could go a long way in filling the knowledge and skill gaps related to nutrition. It is expected that following the incorporation of nutrition in the training manuals of Community Health Workers (CHWs) [23] and other health professionals such as the CHOs [13] with the objective of improving nutrition in children and mothers, the delivery of maternal and child nutritional services at the CHPS zones will improve remarkably.

A major challenge to the provision of maternal and child nutrition services in the Jirapa municipality were lack of financial support, motivation or incentives for the CHWs. The finding in this study is consistent with that of other studies that looked at challenges facing CHOs and CHWs under the CHPS concept in Ghana [21, 24, 25]. Currently Ghana does not remunerate its CHWs contrary to recommendations by the WHO and existing empirical findings in the literature which suggests that there are better health outcomes at the community level when CHWs are remunerated [26]. A previous intervention in Ghana that provided incentives as a means of motivating CHWs and ensuring accountability towards improving maternal and child health and nutrition indicated a positive impact on some maternal health outcomes such as ANC visits although the impact was not statistically significant [4]. Our finding also adds to the growing literature on the need for CHWs to be remunerated in whatever way, if specific services such as maternal and child nutrition services are to be improved at the CHPS level. It is also worth noting how supporting CHWs with non-monetary incentives such as soap and T-shirts amongst others can go a long way in improving general health and nutrition specific services at the community level. For example the issue of supplying CHMCs with T-shirts as a way of identifying them in the communities served as a major motivator to this category of CHWs in this study.

This study revealed the importance of supervisory support visits by the Municipal Health Management Team (MHMT) and its potential impact on improving maternal and child nutrition services. Earlier studies in the Upper East [27] and Upper West [28] regions of Ghana demonstrated improved productivity amongst CHOs who had effective supervision visits from their District Health Management Teams (DHMTs). One of the reasons that necessitated the review and re-launch of the CHPS policy in 2016 by the Ministry of Health (MoH) was to address the challenge of inadequate or lack of supportive supervision resulting in a policy directive on supervision, monitoring and evaluation in the new CHPS policy. This policy directive requires the district director of health services to have overall responsibility for guiding service delivery in the CHPS zones in the district. It also requires a direct supervision of CHOs to be the responsibility of the officer in charge of the health centre in the sub-district. Furthermore, medical officers in the district hospital should be assigned a number of sub-districts for which they shall have mentoring and technical supervision responsibility which includes visiting CHPS zones in their assigned sub-district at least once every quarter [12]. However the findings of this study indicates that these targets are still not being achieved probably due to inadequate funding and logistics at the levels of the MHD and sub-districts as intimated by some participants.

Regarding the CHPS zones based challenges, this study revealed the lack of effective community engagement, home visits, planning and commitment on the part of CHOs to be major concerns. The concept of home visits is an integral part of the CHPS program which requires CHOs to carry out regular home visits to render PHC services [10–12]. However this important function is constraint in the Jirapa municipality partly due to lack of logistics such as transportation at the CHPS zones and partly as a result of lack of supervision or monitoring from the MHDT. The role of community engagement by CHOs to ensure effective health services delivery at the community level cannot be overemphasized. Previous studies [21, 24] have highlighted how the lack of effective community engagement was identified as a major challenge to service delivery in CHPS zones which is consistent with the findings in this study. The CHOs acknowledged the need for effective community engagements and regular meetings with community members as a formidable strategy for improving maternal and child nutrition services at the community level.

Although planning for the activities at the CHPS zones is often done at the district and sub-district levels, participants opined that some level of planning for their day-to-day operations relating to delivering maternal and child nutrition and general health services was also required at their level. This opinion reiterates one of the implementation challenges of the initial CHPS program which identified that, planning as a process at the community level was inadequate thus necessitating a new policy direction [12].

### Strengths and limitations

This study conducted interviews amongst twelve participants out of a potential thirty six participants for the entire district. Nonetheless, saturation was reached at the eleventh interview and therefore was not necessary to have carried out more interviews. The study also concentrated on only CHOs therefore living out the opinions of the CHVs and CHMCs who work hand-in-hand with the CHOs. Their views too on the subject matter could have enriched the study than just relying only on those of the CHOs.

## Conclusion

The delivery of maternal and child nutrition services at the most basic level of the PHC system which is the CHPS zones, is bedeviled with challenges which are both district or municipal health management and CHPS zone level based. District management level based challenges included inadequate logistics/medicines; lack of staff training; lack of supervision/monitoring; and inadequate financial support/motivation/incentives whilst CHPS zones based challenges were lack of planning activities by staff; inadequate home visits; lack of commitment by staff; and lack of community meetings/engagements. Therefore strategies to improve maternal and child nutrition services are aimed at addressing these challenges at these levels of the primary health care system.

We recommend to the Jirapa MHD and all stakeholders involved in maternal and child nutrition services at the CHPS zones to ensure that adequate logistics/medicines are provided; organize frequent training of staff on maternal and child nutrition related issues; carry out frequent supervision or monitoring activities; and provide financial support/motivation/incentives to CHOs, CHVs and CHMCs at the CHPS zones. We also recommend to the heads of the CHPS zones in the municipality to assist their staff to do the following: properly plan their daily health service delivery activities; improve home visits; and organize frequent community meetings or engagements. We further recommend that any future initiative or intervention aimed at improving maternal and child nutrition services at the community level should incorporate the above recommendations including actively engaging CHOs as key stakeholders in order to ensure that such interventions are successful.

### Electronic supplementary material

Below is the link to the electronic supplementary material.

Supplementary Material 1

## Data Availability

All relevant data and information based on which conclusions are made can be found in this write up. The raw de-identified data may be made available upon reasonable request from the corresponding author.

## Abbreviations

CHPS: Community-based Health Planning and Services
CHO: Community Health Officers
CHV: Community Health Volunteer
CHMC: Community Health Management Committee
CHW: Community Health Worker
DHD: District Health Directorate
DHMT: District Health Management Team
GHS: Ghana Health Service
KII: Key Informant Interviews
LMIC: Low and Middle-Income Countries
MCHNP: Maternal and Child Health and Nutrition Improvement Project
MCSP: Maternal and Child Survival Program Ghana
MHD: Municipal Health Directorate
MoH: Ministry of Health
MHMT: Municipal Health Management Team
NNP: National Nutrition Policy
ORS: Oral Rehydration Salt
PHC: Primary Health Care
SDG: Sustainable Development Goals
UHC: Universal Health Coverage
WHO: World Health Organization
