# Supplementary material for: Improving maternal and child nutrition services in community based health planning and services zones in the jirapa municipality of northern ghana-challenges and strategies: the perspective of community health officers
Source: BMC Nutr. 2024 Jun 14;10:87. doi: 10.1186/s40795-024-00848-8 (PMC11179358; doi:10.1186/s40795-024-00848-8)
Supplement: Supplementary file 1 — Supplementary Material 1 [file 40795_2024_848_MOESM1_ESM.docx]

**IN-DEPTH INTERVIEW GUIDE**

**Challenges and strategies to strengthen and improve maternal and child health and nutrition activities in CHPS zones in Jirapa Municipality**

INTERVIEW WITH NUTRITION OFFICER AT THE MUNICIPAL HEALTH DIRECTORATE

1. What are some of the challenges associated with rendering maternal and child nutrition services in the Jirapa Municipality?
2. What measures do you think should be put in place to improve on maternal and child nutrition in the CHPS zones?

INTERVIEW WITH COMMUNITY HEALTH OFFICERS (CHOs)

1. What are some of the challenges associated with rendering maternal and child nutrition services in the Jirapa Municipality?
2. What measures should be put in place by the district or municipal health directorate to improve maternal and child health and nutrition care in the CHPS zones?
3. What should the community health officer (CHO) personally do to improve on maternal and child nutrition care services in the CHPS zones?
4. What do you recommend be put in place to enable community health volunteers contribute better towards maternal and child health and nutrition care service delivery in the communities?
5. What do you recommend be put in place to enable community health committee members contribute better towards maternal and child health and nutrition care service delivery in the communities?
